# Recent knowledge concerning mammalian sperm chromatin organization and its potential weaknesses when facing oxidative challenge

**DOI:** 10.1186/2051-4190-24-6

**Published:** 2014-04-01

**Authors:** Anais Noblanc, Ayhan Kocer, Joël R Drevet

**Affiliations:** GReD Laboratory, CNRS UMR 6293 - INSERM U1103 - Clermont Université, Aubière, France

**Keywords:** Spermatozoa, Protamines, Histones, Sperm DNA integrity, DNA oxidative damage, Spermatozoïdes, Protamines, Histones, Intégrité du noyau spermatique, Dommage oxydant à l’ADN

## Abstract

Spermatozoa are the smallest and most cyto-differentiated mammalian cells. From a somatic cell-like appearance at the beginning of spermatogenesis, the male germ cell goes through a highly sophisticated process to reach its final organization entirely devoted to its mission which is to deliver the paternal genome to the oocyte. In order to fit the paternal DNA into the tiny spermatozoa head, complete chromatin remodeling is necessary. This review essentially focuses on present knowledge of this mammalian sperm nucleus compaction program. Particular attention is given to most recent advances that concern the specific organization of mammalian sperm chromatin and its potential weaknesses. Emphasis is placed on sperm DNA oxidative damage that may have dramatic consequences including infertility, abnormal embryonic development and the risk of transmission to descendants of an altered paternal genome.

## Résumé

Le spermatozoïde est la cellule la plus petite et la plus cytologiquement différenciée chez les mammifères. D’une apparence proche de celle d’une cellule somatique au début de la spermatogenèse, la cellule germinale mâle va, au travers d’un processus hautement sophistiqué, atteindre une organisation finale entièrement dédiée à sa mission qui est de conduire le lot chromosomique paternel au sein de l’ovule. Afin de pouvoir accommoder l’ADN paternel dans la minuscule tête du spermatozoïde, un remodelage complet de la chromatine est nécessaire. Cette revue est essentiellement concentrée sur les aspects connus à ce jour de ce programme de condensation nucléaire spermatique. Une attention particulière est donnée aux avancées les plus récentes concernant l’organisation très spécifique du noyau spermatique et sur ses points de fragilité, en particulier face aux dommages radicalaires. Ces derniers peuvent avoir des conséquences dramatiques qui se posent en termes d’infertilité, de développements embryonnaires anormaux et de risque de transmission à la descendance d’un patrimoine génétique paternel altéré.

## Chromatin structure from spermatogonia to spermatozoa

**Figure 1:**
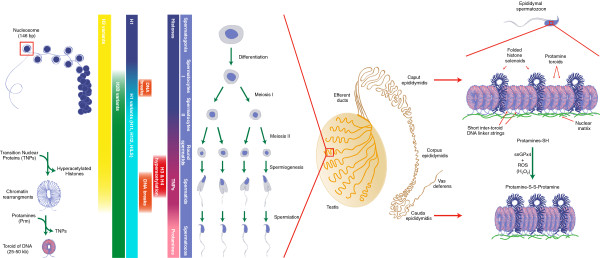
**Schematic representation of the testicular and epididymal events leading to the drastic change in sperm chromatin organization.** During spermatogenesis which takes place within the epithelium of the seminiferous tubule (boxed area in the schematized testis) sequential post-translational modifications of histones occur as well as insertion of testicular-specific histone variants. These events precede the replacement of most histones by transition proteins (TNPs) which in turn at the end of the spermatogenetic program (ie. spermiogenesis) will be replaced by protamines (PRMs). These modifications allow for the compaction of the majority of the sperm chromatin in toroidal structures each embedding 50 to 100 kb of DNA. thus permitting the great decrease in nuclear volume (one tenth that of a somatic nucleus). At the end of spermatogenesis a fraction of the sperm chromatin is still in nucleosomal arrangement. Remaining histone-containing nucleosomes (folded histone solenoids) punctuate the toroidal chromatin structure. In addition, the small DNA linker strands going from one toroid to another are also associated with histones. At some points, these histone-associated strings of DNA are bound to the sperm nuclear matrix [63]. During post-testicular epididymal maturation of spermatozoa, the nucleus is further condensed by means of intense disulfide bridging. A nuclear located enzyme (sperm nucleus glutathione GPx4 = snGPx4) working as a disulfide isomerase uses luminal reactive oxygen species (ROS), essentially hydrogen peroxide (H_2_O_2_) to create inter- and intra-protamine disulfide bounds on thiol groups carried by the cysteine-rich protamines. It further condenses the sperm nucleus and locks it up in that condensed state [93].

Spermatozoa are the result of spermatogenesis, a process generally divided into three phases (if one excludes the spermiation process) which takes place in the seminiferous tubules of the testis. In the first phase, primitive germ cells or spermatogonia undergo a series of mitotic divisions. In the second phase, spermatocytes go through two consecutive meiotic divisions to produce the haploid spermatids. In the third and last phase, spermiogenesis, spermatids differentiate into highly polarized spermatozoa cells with extensively modified chromatin compared with the germ cell. In spermatogonia and spermatocytes, germinal cell chromatin is identical to that of somatic cells. It consists in a combination of DNA associated with small basic nuclear proteins, the histones. These proteins are rich in lysine and arginine residues, giving them a global positive charge allowing their interaction with the negatively charged DNA in a well-organized structure known as the nucleosome (see Figure 1). One nucleosome, is composed of 146 base pairs (bp) of DNA wrapped in 1.67 turns around a histone octamer consisting of two copies of the histone core proteins H3, H4, H2A and H2B. The full length of the DNA molecule is associated with these core nucleosomal particles and acquire a *beads on a string* structure. A fifth histone, H1, interacts with a linker DNA sequence connecting two nucleosomes allowing greater compaction of the chromatin. The histones organize the chromatin as a fiber of 11 nm in diameter, which coils itself into a larger and shorter fiber that will fit in the tiny nuclear compartment.

The structure of somatic chromatin is not homogenous. A somatic nucleus observed by transmitted electronic microscopy (TEM) presents areas of variable density depending on the level of chromatin compaction. Clear, less condensed areas in the center of the nucleus correspond to euchromatin, which is more accessible to protein complexes involved in transcription and contains active genes. Dark, more condensed areas at the nuclear periphery called heterochromatin, contain transcriptionally repressed genes essentially because they are not accessible to the transcriptional machinery. However, these nuclear areas are variable between cell types to another, and also depending on cell differentiation levels. Transition between euchromatin and heterochromatin results from modifications of the physic-chemical properties of histones and DNA. These changes alter interactions between these two components and relax or condense the chromatin not only to regulate gene expression, but also to allow DNA repair, DNA replication, mitosis, and meiosis. One of these processes consists in enzymatically-controlled post-translational modifications (PTM) of histones, occurring principally on their amino-terminal tail protruding from the core nucleosome. Different PTM including acetylation, methylation, phosphorylation, and ubiquitinylation have been identified and extensively studied. Taking methylation as an example, the same histone can be modified on different residues at the same time and, a chemical group can be added up to three times on the same residue leading to dimethyl or trimethyl variants. Most of these modifications are reversible, giving great plasticity to chromatin and allowing cells to react and to adapt efficiently to their environment. All these modifications are referred to as the histone code [1, 2].

To modify DNA-nucleosome interaction efficiently, the most drastic way is probably to exchange a canonical histone with another protein, a histone variant [3]. To each canonical histone correspond different histone variants, which are homologous proteins of the same family encoded by distinct genes. Sequence identity between a variant and its corresponding canonical histone varies. For example, H3 shares 96% identity with the H3.3 variant and only 46% identity with the centromere-specific protein A (CENP-A), another H3 variant. These modifications in amino acid sequence confer to histone variants specific structures as well as their own physic-chemical properties. Thus, histone variants possess different biological functions when compared with canonical histones. Interestingly, several of these histones variants were found to be testis-specific and solely expressed in germinal cells during spermatogenesis.

### Chromatin remodeling in germ cells

During spermatogenesis, germ cells undergo a long process of differentiation to form spermatozoa, highly cyto-differentiated cells constituted of a head containing the nucleus, the paternal genetic material transmitted at fertilization, and a flagellum allowing them to move up the female genital tract to encounter the female gamete, the oocyte. The passage from a spermatogonia, a diploid cell, to four haploid cells called spermatids, results from meiosis. As for mitosis, this process requires chromatin modifications in multiple steps in order to separate homologous chromosomes and chromatids in identical sister cells. This remodeling of chromatin during meiosis is permitted by histone PTM and by insertion of ubiquitous and/or testis-specific histone variants in multiple steps including chromatid condensation, repair of the numerous DNA single strand breaks (SSB) needed for homologous chromosome pairing, sex (or XY) body formation, massive activation of transcription during the pachytene stage, and formation of kinetochore thanks to the CENP-A H3 variant (see Figure 1). The precise functions of all these chromatin modifications during meiosis are still under study (for reviews see: [4–6]). We present here the most critical stage of these events for DNA integrity preservation and genetic diversity: prophase I.

The long prophase I is divided into five phases (leptotene, zygotene, pachytene, diplotene and diakinesis) which consist in pairing of homologous chromosomes allowing exchange of genetic information between them, by formation of the synaptonemal stabilized protein complex, and of double strand DNA breaks (DSB). At the beginning of meiosis, spermatocytes undergo replication of their genome during which testis-specific histone variants (TH2B) start to be incorporated. At the end of the pre-leptotene phase, all the chromosomes are composed of two sister chromatids linked by a protein complex called cohesin, which is important for chromosome segregation and DNA repair *via* homologous recombination [7, 8]. During the leptotene phase, chromatin condensation occurs through histone deacetylation and methylation. This methylation step also appears to be involved in the control of homologous chromosome pairing by an unknown mechanism (control of chromatin conformation to drive DSB localization and/or regulation of meiosis-implied gene transcription) [9]. Moreover, DSB occur all along chromatids, inducing local insertion of the histone variant γH2A.X and hyperacetylation of H4 to open the chromatin and prepare DNA repair [10]. The zygotene phase starts when the axial element of the synaptonemal complex is formed all along sister chromatids. Homologous chromosomes then pair up by recognizing free homologous double stranded DNA. This labile association allows the formation of the final synaptonemal complex between homologous chromosomes. This stable association permits DNA repair in numerous DSB by homologous synthesis without crossover, greatly reducing the quantity of γH2A.X. The next step of the prophase I is the pachytene stage, when homologous recombination with the formation of crossovers occur, permitting genetic information exchange between homologous chromosomes and the repair of the last DSB. Simultaneously, the incorporation of new histone variants takes place (THA2, TH2B, H1t, H3.3) and opens the chromatin to allow for massive transcription. These chromatin modifications are facilitated by the ubiquitinylation of H2A throughout the genome and the acetylation of H3K9. During this stage, in contrast the sex chromosomes undergo condensation which inactivates them, and forms the sex (XY) body. When all DSBs are repaired, the diplotene stage starts: the synaptonemal complex is dismantled and chimeric homologous chromosomes are only joined by *chiasmata*, at the crossover sites, ready to be separated by the mitotic spindle during the end of meiosis I.

Besides these early germ cell chromatin modifications, at the end of the spermatogenetic program (i.e. during spermiogenesis) the male nucleus is subjected to deep structural modifications. During this ultimate phase, paralleling drastic changes in cell size and morphology when spermatids are compared with spermatozoa, the nucleus is greatly reduced (approximately by 10 fold) to fit it into the smallest possible volume. This extreme compaction serves two major goals. It allows the acquisition of a more hydrodynamic head shape that will directly help speed of movement, l and, in addition, it protects paternal DNA from genotoxic influences. The drastic modification of chromatin conformation during spermiogenesis winds and ties the DNA molecules and eventually breaks DNA strands. Paradoxically, to protect the DNA from such damage, topoisomerase enzymes cut and re-anneal DNA strands in a controlled way to relax and unknot the DNA during chromatin remodeling [11, 12]. In the nucleus of elongating spermatids, between 5 to 10 million DNA breaks occur and are repaired during chromatin remodeling [12, 13]. All these DNA breaks constitute a possible source of heritable genetic mutations in case of defective repair [14].

Sperm chromatin condensation is achieved by a profound and sequential reorganization of DNA-associated proteins. Briefly, at first different histone modifications (such as extensive acetylation) as well as incorporation of histone variants — in particular, linker histone variants: H1t, H1t2, and HILS — take place and open the chromatin to facilitate exchange between histones and new proteins, the transition proteins (TNP). Second, TNP are replaced by other proteins, the protamines (PRM).

Among histone PTM occurring during spermiogenesis, simultaneous hyperacetylation and ubiquitinylation seem to play an important role in the histone-PRM exchange. H2A and H2B ubiquitinylation adds a large chemical group to the core histone that causes a steric hindrance, opening the chromatin. In the same time, histone deacetylases (HDAC) are degraded [15], causing hyperacetylation of H4 and H3 in the entire nucleus. In human, this hyperacetylation consists of an acetylation sequence of multiple histone residues in a defined order that precedes and persists during histone-to-PRM exchange. This process of histone hyperacetylation in male germ cells only occurs in species concerned by the histone replacement (trout, mollusks, *Drosophila*, rooster, rodents, human), and is weaker in species such as monotremes/marsupials, conserving more histones in the mature sperm cells. Two modes of action of the histone hyperacetylation have been proposed and are not mutually exclusive. First, DNA-histone interaction is decreased by histone hyperacetylation, opening chromatin and allowing recruitment of factors and protein exchange. Second, bromodomain proteins recognize and bind hyperacetylated histones. One of these proteins is the bromodomain testis-specific protein (BRDT), [16, 17]. Binding of BRDT to hyperacetylated H4 induces chromatin condensation, independently of ATP, perhaps affecting structure [17, 18]. BRDT binding allows the recruitment of SMARCE1 [17], an ATP-dependent SWI/SNF chromatin remodeling complex, which suggests a double mechanism of action of BRDT *via* ATP-dependent and ATP-independent processes.

### The replacement of histones by transition proteins (TNP)

In mammals, hyperacetylated histones are first replaced by transition proteins, which is not the case in all species such as for example in some mollusks, where histone-PRM exchange does not need an intermediary [19]. TNP are small proteins (between 50 and 140 residues) more basic than histones, but still less than PRM, because they are rich in arginine and lysine. Four TNP are known and amongst them only TNP1 and TNP2 have been well studied. TNP1 and TNP2 are encoded by two different single-copy genes composed of 2 exons and an intron. In rodents and humans, *tnp2* is included in a gene cluster with *prm1*, *prm2*, and *prm3*. This gene cluster is surrounded by 2 matrix attachment regions (MAR), which are involved in transcriptional regulation of these genes during spermiogenesis [20]. The transcription of these clustered genes and *tnp1* occurs at the same time in round spermatids and their corresponding mRNAs are stored as ribonucleoproteins. Subsequently, Tnp mRNAs are translated and TNP proteins are phosphorylated on the C-terminus, a prerequisite allowing DNA binding. It is ultimately removed to increase TNP-DNA affinity and chromatin condensation [21]. A highly regulated transport of transition proteins into the nucleus ensures their availability. It was shown that the phosphorylation of TNP2 modulates its nuclear import [22] and that the importin β4 was involved in this nuclear transport [23].

TNP1 protein is 54 amino acids long, composed of 20% lysine, 20% arginine, and no cysteine — except in boars, bulls, and rams — in a highly conserved sequence between species. This protein is strongly expressed and homogeneously distributed in the nucleus of spermatids. *In vitro*, TNP1 decreases the melting temperature of DNA [24], destabilizes nucleosome-DNA interaction and relaxes chromatin when it is added to nucleosome-bound DNA [25]. TNP1 also increases topoisomerase I activity [26] and stimulates single-strand break repair [27]. *In vivo*, *tnp1* knock-out in mice did not induce a marked phenotype in sperm nucleus, but nevertheless was associated with an infertility [28]. Only 40% of male mice were fertile and litter sizes were reduced from 7.7 to 1.6 when males were mated with females of the same background. It was proposed that this infertility was a consequence of greatly decreased sperm motility. In spermatid nuclei, an abnormal chromatin structure was observed during condensation with the presence of rod-shaped chromatin condensation units in the fine fibrillar chromatin. Moreover, chromatin of epididymal mature *Tpn1*^*-/-*^ spermatozoa was less condensed than in wild-type (WT) mice. The study of protein composition in spermatid nuclei revealed normal histone withdrawal but increased incorporation of TNP2 and premature production of the precursor of PRM2 protein. Furthermore, processing of the PRM2 precursor by cleavage was delayed and persisting PRM2 intermediate was observed in cauda epididymal spermatozoa.

TNP2 is relatively different from TNP1 in many aspects. This protein is twice as large as TNP1, with a 117–138 amino acids poorly conserved sequence between species. It is composed of 10% lysine, 10% arginine, 5% cysteine, as well as serine and proline. TNP2 possesses 2 zinc-finger domains in the N-terminal region and a highly basic C-terminus. Its expression level varies depending on species. *In vitro*, TNP2 increases the melting temperature of DNA and condenses nucleosome-bound DNA by oligomerization of close DNA strands [29, 30]. *In vivo*, *Tnp2*-null mice were fertile, even if a decrease in the litter size was reported from 7.4 to 3.9 pups per litter [31]. Epididymal spermatozoa presented flagellar defects and an abnormal chromatin structure, similar to that observed in *tnp1*-null mice and less condensed when compared to WT mice. Moreover, compensation of TNP2 loss was achieved by an increase in TNP1 expression and the same maturation defect of the PRM2 precursor as in *tnp1*-null mice was observed.

*Tnp1*/*tnp2*-null double mutant mice were also generated [32]. These mice were infertile and showed a large decrease in epididymal sperm count, motility and viability, associated with abnormal sperm morphology and defects in chromatin condensation. *In vitro* fertilization assays with these spermatozoa revealed weak fertilizing abilities. *In fine*, these studies underlined that TNP1 and TNP2 possess some redundant functions, but cannot fully compensate for one another, suggesting individual functions as well. The opposed *in vitro* properties of TNP1 and TNP2 also support these conclusions.

### The replacement of TNP by PRM

During spermiogenesis transition proteins are readily replaced by PRMs. Only PRM1 and PRM2 were characterized in mammals. If PRM1 is expressed by all mammals, PRM2 is only expressed in some species including primates, some rodents, rabbits, hares, and horses. Although pigs and bulls possess a *prm2* gene, it is not functional in these species. PRM1 and PRM2 genes are composed of 2 exons and an intron, similarly to *tnp* genes. As indicated above, in rats, mice, and humans, they are parts of a cluster with *tnp2* and *prm3*. These *prm* genes are expressed at the same time in round spermatids and the corresponding mRNAs are stored (for reviews see: [21, 33, 34]. It should be noted that *prm3* encodes a small cytoplasmic acidic protein, not involved in spermatid chromatin condensation [35]. Similarly to TNPs, PPRMs are phosphorylated immediately after mRNA translation, during translocation of proteins into the nucleus. This PTM allows DNA binding. Its removal increases the PRM-DNA affinity and chromatin condensation.

PRM1 is translated as a mature protein of about 50 amino acids, composed of an arginine-rich central domain and cysteine-rich short domains. The N-terminal tail possesses serine residues which are concerned by the phosphorylation events as indicated above. PRM2 is synthesized as a precursor protein of a hundred amino acids. Poly-arginine domains are interspersed throughout mature PRM2 and its content in histidine is higher than in PRM1 [36]. As for PRM1, PRM2 contains numerous cysteine residues. It is also phosphorylated immediately after its synthesis, enabling it to bind to DNA. DNA-binding PRM2 is progressively matured by successive proteolytic cleavages of its N-terminus over several days, increasing step by step chromatin condensation. This maturation process removes about 40% of the N-terminal domain of PRM2. In mice and humans, 6 cleavages are necessary to produce a mature protein about 60 residues long. It is interesting to note that, some of the intermediate products can persist in the mature sperm nuclei [37, 38]. Another important difference between PRM1 and PRM2 is the ability of PRM2 to bind zinc, which allows it to bind to DNA.

### Final structure of sperm chromatin

Histone-PRM exchange during the elongating phase of spermiogenesis modifies drastically the structure and spatial organization of sperm chromatin, which becomes 10 times more condensed than in somatic cells. However, sperm chromatin must be highly ordered in the small spermatozoa nuclear space to allow its rapid decondensation upon fertilization and an immediate use of the paternal genome by the zygote. Even if this organization is still not completely understood it has been largely studied during the last decades.

### The basal unit of sperm chromatin and its conformation

The nucleoprotamines are the basal units of sperm chromatin. As shown by raman spectrometry, when PRM1 is free in solution, the protein is unfolded [39]. PRM1 acquires a stable conformation only when it is bound to DNA; wrapping around the double stranded DNA, in one groove of the double helix *via* electrostatic and hydrogen bonds with the DNA backbone. The interaction of one protamine per turn helix (~11 bp for PRM1, [40]) curves the DNA and gives a new conformation to sperm chromatin. After this binding, intra-molecular disulfide bridges are first formed to stabilize the PRM1-DNA interaction and, then, intermolecular disulfide bridges are made between Prms to associate and bring adjacent DNA fibers closer, condensing the sperm chromatin. Similar properties were found concerning PRM2-DNA interaction. However, it did appear that the zinc ion is here involved in this interaction. These observations have led to the proposal that, if PRM1s are linked by intermolecular disulfide bridges between their cysteine residues, PRM2s might be linked or stabilized by zinc bridges [41]. Atomic force microcopy studies revealed that the addition of bull PRM1 to a free linearized plasmid DNA on a mica surface permitted its condensation into a toroidal subunit [42]. The diameter of these toroids was about 40 nm with each coil consisting of ~360–370 bp. Other *in vitro* experiments using salmon protamine showed that around 50 kb of DNA can be coiled into a toroid [43]. Toroids were also observed in native human sperm chromatin [44].

In the last twenty years, the study of mammalian sperm chromatin confirmed and completed these *in vitro* data. In high salt conditions, PRM can be extracted from the nucleus of epididymal spermatozoa by the reduction of the disulfide bridges between PRM using a reducing agent such as dithiothreitol (DTT). This treatment induces the formation of a halo composed of DNA loops around the sperm nucleus. This halo is visible after staining by ethidium bromide and its measurement indicated an average DNA loop length of 46 kb in hamster spermatozoa [45]. The same experiment on human sperm cells revealed a DNA loop size of about 27 kb [46].

#### Sperm nuclear matrix

The formation of a DNA halo around the sperm nucleus after protamine extraction suggested that the DNA loops correspond to DNA free from toroids but still attached to an internal nuclear structure. Ward and colleagues proposed that the toroid extremities are associated with a protein-nuclear matrix. They also demonstrated that the DNA strand bridging one toroid to another is sensitive to nucleases, as is the case with nuclear matrix attachment regions (MAR) in somatic cells [47]. Moreover, in the sperm nucleus of hamsters, mice, and humans, they isolated a protein structure which is bound to specific DNA sequences and is a part of the nuclear matrix [46, 48, 49]. This structure was called the nuclear annulus because of its curved ring shape. It was located at the base of the sperm nucleus at the implantation fossa, the junction of the sperm midpiece structure to the sperm head. Further studies of sperm MARs demonstrated their cell type specificity. Additional research on mice underlined the importance of physical association between these DNA sequences and the sperm nuclear matrix for paternal pronucleus formation and the first DNA replications in the zygote [50, 51].

#### Sperm persistent nucleosomes

Recently, it was shown that sperm MARs are not associated with PRMs, but rather are enriched in persistent histones [52]. It was estimated that 1% to 2% histones persist in mice, hamster, stallion and bull sperm nuclei [40, 53] while up to 10% remains in human sperm nuclei [54]. These histones also appear to persist in the zygote [55], after the PRM-histone exchange occurring 4 hours after fertilization [56, 57]. Thus, sperm persistent histones are part of the paternal inheritance that may play a significant role in early embryo development [58, 59].

As demonstrated by genome-wide analyses (chromatin immuno-precipitation, DNA microarray, high-throughput sequencing) persistent histones are not randomly distributed, suggesting that their persistence is not a consequence of a histone replacement defect and that they are not simply remnants of the sperm differentiation program [60, 61]. Nucleosomes were found enriched in 2 types of sperm genomic regions: in large areas of DNA up to 100 kb that punctuate the compacted toroid-organized chromosomes and, in short DNA linker sequences going from one toroid to the other. This last location was proposed to correspond to the highly sensitive short DNA sequences, attached to the sperm nuclear matrix, in between toroids [62]. It was also suggested that the larger histone-bound regions could be organized in a less condensed chromatin, with a conformation closer to the typical solenoid of somatic chromatin.

Gene ontology analyses revealed that persistent histones were significantly enriched at promoters of genes coding for microRNAs, genes involved in early embryonic development (such as genes encoding transcription factors and/or signaling proteins…), genes subjected to genomic imprinting, and genes involved in spermatogenesis [61]. A detailed analysis of some histone variant distributions in these loci revealed that testis-specific H2B variant (TH2B) was found enriched with promoters of genes involved in sperm cell maturation, capacitation and fertilization, but never with promoters of genes controlling embryonic development. The H2A.Z variant was essentially found in peri-centromeric heterochromatin. Concerning the PTM of canonical histones, the promoters of genes encoding developmental transcription factors, were found enriched in H3K4me2 marks while H3K9me3 marks were not localized near genes, but rather in peri-centromeric genomic regions [61].

The spatial organization of the histone-rich DNA sequences in the nucleus was also not found to be random. In mice and humans, immunological detections of canonical and variant histones demonstrated that these proteins were localized mainly at the periphery of the sperm nucleus and in the nuclear post-acrosomal basal domains [62–64]. This second localization encompasses the structure called the nuclear annulus, characterized by Ward and Coffey [48]. It is seen as a component of the sperm nuclear matrix, acting as an anchor for sperm DNA. It is viewed as a structural organizer of the sperm chromatin, *via* the MARs and the histone-rich telomeres [63, 65]. Recently, the observation that there is an interaction between histone-bound DNA and the sperm nuclear matrix was strongly reinforced by the demonstration of partial co-localization of sperm-persisting histones with topoisomerase IIβ, a protein marker of MAR-attached sequences [64].

#### Sperm chromosomal organization

Further studies also demonstrated the highly conserved sperm chromatin organization in chromosomes, between cells and individuals of the same species. In the last two decades, the FISH technique applied to sperm cells revealed that the chromosomes are non-randomly positioned in the sperm nucleus. In humans, it appeared that chromosomal centromeres are mostly localized in the center of the sperm nucleus, whereas telomeres are preferentially at the periphery [66]. The use of FISH probes for each arm of one chromosome showed that the q- and t-arms co-localized in the same limited territory of the human sperm nucleus. This led to the proposal that the two arms of a chromosome interweave or juxtapose in an antiparallel fashion, such that each chromosome has a hairpin structure on a center-periphery axis [67]. Individual chromosomes were found to be non-randomly localized in relation to each other. They occupy a precise position in the sperm nucleus and are not intertwined. For example, according to statistical studies, the relative localizations of autosomal and sex chromosomes are maintained between sperm cells of an individual and between individuals of one species [68–70]. In humans, the organization of some chromosomes (17, 1, X, 19, Y) was partially established along the anterior-posterior axis of the sperm nucleus [70, 71]. Furthermore, the observation of chromosome positioning in some rare diploid spermatozoa (~0.2%) showed that this order was the same between the two sets of chromosomes in a given nucleus and between haploid and diploid sperm cells suggesting that the chromosomal organization observed in sperm cells is established during meiosis [70].

### How and why sperm chromatin can be affected?

Sperm as well as oocyte chromatin integrity is an important factor conditioning reproductive performance including fertilization success rate, successful completion of the developmental program, quality of life of the offspring, and, overall, species persistence [72–74]. Even if the sperm chromatin is packaged in a highly compacted state, the spermatozoa nucleus is still vulnerable and can suffer damage. There have been many reports showing that sperm DNA alterations can compromise the reproductive outcome both in natural conception and in ART by interfering with normal embryo development, increasing the risk of morbidity in the offspring as well as the development of diseases such as childhood cancer, progeny infertility and the occurrence of spontaneous dominant genetic diseases such as achondroplasia or Apert syndrome [75, 76]. In addition, it has been shown that sperm chromatin damage was associated with abnormal chromatin de-condensation patterns during the initiation of pronucleus formation after fertilization [52].

**Figure 2:**
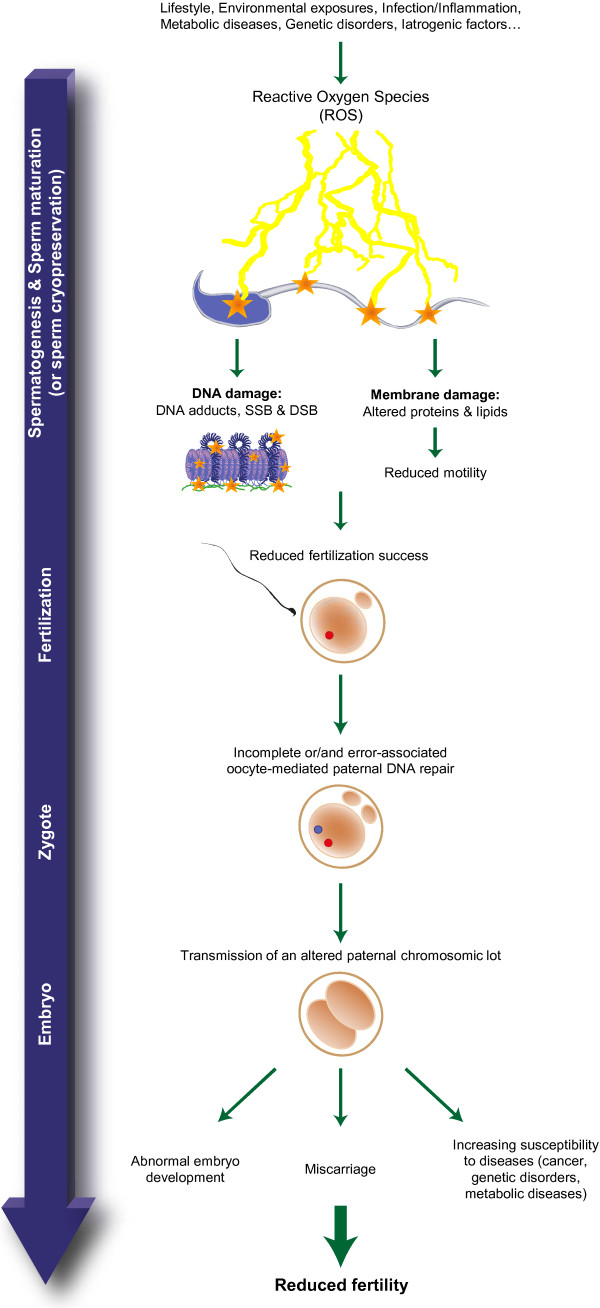
**Reactive oxygen species damaging effects on spermatozoa and its consequences.** Reactive oxygen species provoke membrane and nuclear alterations on spermatozoa resulting in reduced motility, reduced fertilization ability and the risk of transmission to the progeny of an altered paternal chromosomal lot if it is not properly repaired by the oocyte following fertilization. Alternatively, *de novo* mutations can also be introduced during the repair process when too many oxidized bases have to be replaced within the paternal pronucleus. Eventually, this altered chromosomal lot may be at the origin of abnormal embryo development, miscarriage, perinatal/postnatal mortality and an increased susceptibility to diseases for the young or/and the adult.

Most sperm DNA damage, in particular either single or double DNA strand breaks, are inherent to the spermatogenetic process itself essentially because of meiotic errors and the mechanical constraints described above accompanying nuclear histone replacement and DNA condensation. Although it is yet unknown whether breakage is mechanical, enzymatic or ROS-induced it is however admitted that the most common sperm DNA alteration is oxidative damage leading to the formation of oxidized bases such as the 8-OHdG (8-hydroxy-2’-deoxyguanosine) residue. As very recently reviewed in [77, 78] oxidative damage to sperm DNA is now considered one of the most important causes of defective sperm functions. Sperm DNA oxidative damage (SDOD) (Figure 2) was associated with defective spermatozoa and decreased fertilizing potential [79–86]. In addition, SDOD was associated with poor fertilization rates, impaired embryonic development, pregnancy loss and birth defects [87–89]. Concurring with these reports, we, and others have shown that a decrease in antioxidant protection in the male reproductive tract is particularly critical for spermatozoa functions [85, 88].

SDOD occurs during situations of oxidative stress, which are very frequent and may be physiological or non physiological. SDOD was associated with leucocytospermia accompanying inflammation and infection and also with genetic or metabolic disorders such as dyslipidemia. In addition, SDOD was associated with environmental exposures to chemical (ie. polluants/toxicants, drugs, medicines, tobacco smoking…) or physical stressors (ie: heat, ionizing radiations, microwaves,….) [90–92]. Furthermore, SDOD is also a classical consequence of assisted reproductive technologies where spermatozoa are collected, cryo-conserved, cultured and manipulated *ex-vivo*. Hence, it is now admitted that SDOD may well explain the high rate of failure with ICSI using cryopreserved spermatozoa.

Testicular germ cells are at a lower risk of DNA oxidative injury than post-testicular spermatozoa. This can be explained by the fact that there are DNA repair processes in the testis as well as apoptotic disposal of dying cells. The highly sealed seminiferous epithelium also contributes to the protection of differentiating germ cells from blood-vehicled environmental hazards. In contrast, the post-testicular life of spermatozoa is a risky period for accumulation of SDOD. Compared with the testis compartment, the male accessory organs are less sealed environments meaning that spermatozoa have greater chances to be exposed to systemic hazards. Spermatozoa susceptibility to post-testicular SDOD is increased by the fact that mature sperm cells leaving the testis are silent cells with no capacity to elicit stress responses in order to defend themselves from any type of aggressors. The haploid and highly compacted quasi crystalline sperm nucleus forbid transcription while, the near absence of cytoplasm leave spermatozoa with little resource for translation as well as little content in cytosolic protective effectors such as antioxidants. Post-testicularly, spermatozoa will have to rely on their environment for their protection. Should they face pro-oxydant situations they may accumulate SDOD that will not be dealt with by classical DNA repair mechanisms. It was recently shown that only the first step of the DNA repair pathway involving the OGG1 protein occurred in spermatozoa [93]. The second step involving the APE1/XRCC1 proteins of the base excision repair (BER) pathway being completed in the oocyte post-fertilization.

Although the epididymis provides considerable protection for maturing sperm cells, it also puts them in a rather paradoxical situation when it comes to oxidative stress (reviewed in [94]). Briefly, spermatozoa descending the epididymis encounter a pro-oxidant environment that participates in their post-testicular maturation program (reviewed in [95]). Especially, it promotes disulfide bridges on various sperm thiol-containing proteins including protamines. This redox-mediated bridging activity contributes to further compaction of the sperm nucleus as well as locking it in a condensed state, thus participating in the protection of paternal chromosomes. Maintenance of a correct equilibrium between beneficial sperm oxidation and detrimental sperm oxidation in the epididymis relies on an armada of antioxidants effectors from small metabolites to enzymes. Among them, the glutathione peroxidase family occupies a central position [94–98]. If spermatozoa nuclear compaction is not optimal when entering the epididymis or/and if anything happens to challenge the redox equilibrium of the epididymis luminal environment, the long periods of epididymal transit and storage may represent challenging moments when spermatozoa could be at risk of SDOD [95, 97]. Therefore, when dealing with sperm presenting defects in testicular nuclear condensation (for example: defective protamination) ART success rate are expected to be best with testicular sperm than with epididymal sperm.

In 2009, we reported that the deletion of the glutathione peroxidase 5 (GPx5), a primary antioxidant enzyme largely secreted by the mouse caput epididymidis, led to SDOD and fragile sperm chromatin condensation [85]. These sperm nuclear defects were accompanied by higher rates of miscarriage, abnormal development and perinatal mortality in the offspring when *gpx5*-deficient males were mated with WT fertile females and compared with the reproductive issues of a similar number of WT-males mated with WT females in identical conditions [85]. Fertilization did not seem to be significantly affected in these natural crosses [85]. These data confirmed that oxidative alterations of the male nucleus could escape oocyte repair processes and be the cause of reproductive failures. More recently, using *gpx5*-KO spermatozoa we reported that SDOD affects preferentially the regions of sperm nuclei that are still in nucleosomal arrangement (ie histone-associated) [64]. The sperm nuclear domains most susceptible to oxidative damage were found to be at their periphery and at the base of the sperm head where the histones rich-nuclear domains were shown to be associated with the nuclear matrix [52, 64]. Interestingly, these sperm DNA domains were shown enriched in sequences of paramount importance for the early events accompanying the onset of the embryonic developmental program post-fertilization, including initiation and regulation of paternal gene expression, paternal DNA replication origins, sequences involved in the selective activation of developmental genes, imprinted loci, and microRNA clusters [52, 61, 99, 100]. The importance of these particular sperm DNA domains that we found susceptible to oxidation were enforced by observation that intracytoplasmic oocyte injections with isolated sperm DNA devoid of the sequences that remain associated with the sperm nuclear matrix did not permit paternal pronucleus formation and paternal DNA replication [61, 101, 102].

### Concluding comments

It thus appears that the sperm nuclear sequences particularly susceptible to oxidation are of primary importance for the success of reproduction. We strongly support the idea that, for this reason, SDOD should be routinely evaluated in couples having conception difficulties especially when paternal age or/and maternal age are at stake, as well as in any situation where the integrity of the male nucleus is considered not to be optimal. Unfortunately, to date, in routine evaluation of sperm biological parameters very few infertility clinics address sperm DNA integrity. However, in recent years, there has been an emerging worldwide concern among infertility clinicians that sperm nucleus integrity should be better evaluated either using sperm chromatin structure assays (SCSA), sperm chromatin dispersion (SCD), terminal deoxynucleotidyl transferase dUTP nick-end labeling (TUNEL) or the Comet assay (for reviews see: [103–106]). Most of these assays address the question of sperm DNA fragmentation that, if found elevated, was associated with poor prognosis in ART [107, 108]. Although any one, or a combination, of the assays mentioned above could be valuable additions to the routine checklist of sperm quality parameters that would help ensuring the quality of ART outcome, to our opinion it is not sufficient. They should be completed by an evaluation of the level of sperm DNA oxidation. This is supported by the recent observation that approximately 60% of males entering ART protocols present a high level of SDOD irrespective of the origin of their infertility [109]. This indicates that SDOD is a rather common situation in infertile couples that should be monitored. Sperm DNA oxidation is thus a more frequent condition than sperm DNA fragmentation. In fact, if sperm DNA fragmentation is often associated with sperm DNA oxidation, the latter is not necessarily associated with the former. Only when oxidation levels reach very high values it is associated with DNA single and double-strand breaks. However, even with mildly oxidized sperm DNA, post-fertilization when the oocyte will try to repair and to remove all the oxidized bases, the paternal DNA fragmentation will be high and putatively at the origin of errors leading to the introduction of *de novo* mutations. Thus, in our opinion, to improve ART outcome, validation of a powerful clinical assay to evaluate DNA oxidation (such as the 8-OHdG assay) should be the focus of further clinical developments.

## Authors’ information

Dr. Anaïs Noblanc has recently completed her Ph.D in Reproductive Biology at Blaise Pascal University-Clermont2, France. under the supervision of Dr. Ayhan Kocer & Prof. Joël Drevet.

Dr. Ayahn Kocer is Associate Professor at Blaise Pascal University-Clermont2 (France) and a member of the GReD MEPTI’s (Mechanisms of Post-Testicular Infertility) research team since 2009. He completed his Ph.D degree at UVSQ (Université Versailles Saint Quentin, Paris) in 2008 working on mammalian male sex determination and went for a post-doctoral training stay in the group of Dr. Ian Adams in the department « Chromosomes and gene expression » headed by Prof. Wendy Bickmore at the Medical Research Council in Edinburgh (United Kingdom).

Professor Joël R. Drevet, is the leader of the MEPTI’s research group and the adjunct-director of the GReD Laboratory (CNRS research unit UMR 6293-INSERM research unit U1103-Clermont Université) at Blaise Pascal University-Clermont2. Prof. Drevet has editorial duties for *Human Reproduction, PLoS ONE, Andrology, Asian Journal of Andrology, ISRN Urology* and *Basic & Clinical Andrology*. He sits on the board of the French Andrology Society (SALF) and is affiliated with the European Academy of Andrology (EAA), the International Society of Andrology (ISA) and the Society for Study on Reproduction (SSR).

## Abbreviations

8-OHdG: 8-hydroxydesoxyguanosine
APE1: Apurinic/apyimidinic endonuclease 1
ART: Assited reproductive technologies
ATP: Adenosine tri-phosphate
BER: Base excision repair
bp: base pair
Brdt: Bromo-domain testis-specific protein
CENP-A: Centromere-associated protein-A
DNA: Desoxy-ribonucleic acid
DSB: Double strand break
DTT: Dithiothreitol
dUTP: 2’-Deoxyuridine 5’-triphosphate
FISH: Fluorescence *in situ* hybridization
GPx5: Glutathione peroxidase 5
HDAC: Histone deacetylase
H3K9: Histone H3 lysine 9
(H1: H3, H4, H2A, H2B, H2A.Z, H1t, H1t2, HILS, TH2B, γH2A.X): Histone variants
ICSI: Intra-cytoplasmic sperm injection
KO: Knock-out
MAR: Matrix-attachment region
OGG1: 8-oxoguanine glycosylase-1
Prm: Protamine
PTM: Post-translational modification
RNA: Ribonucleic acid
ROS: Reactive oxygen species
SCD: Sperm chromatin dispersion
SCSA: Sperm chromatin structural assay
SDOD: Sperm DNA oxidative damage
SSB: Single strand break
SWI/SNF: SWItch/sucrose non-fermentable nucleosome remodeling complex
TEM: Transmission electron microscopy
Tnp (Tnp1 Tnp2): Transition protein (1 & 2)
TUNEL: Terminal deoxynucleotidyl transferase dUTP nick end labeling
WT: Wild-type
XRCC1: X-ray repair cross-complementing1.
